# *In vitro* cytotoxicity and anticancer effects of citral nanostructured lipid carrier on MDA MBA-231 human breast cancer cells

**DOI:** 10.1038/s41598-018-38214-x

**Published:** 2019-02-07

**Authors:** Noraini Nordin, Swee Keong Yeap, Heshu Sulaiman Rahman, Nur Rizi Zamberi, Nadiah Abu, Nurul Elyani Mohamad, Chee Wun How, Mas Jaffri Masarudin, Rasedee Abdullah, Noorjahan Banu Alitheen

**Affiliations:** 1 Department of Cell and Molecular Biology, Faculty of Biotechnology and Biomolecular Sciences, Universiti Putra Malaysia, 43400 UPM Serdang, Selangor Malaysia; 2 China-ASEAN College of Marine Sciences, Xiamen University Malaysia, Sepang, Malaysia; 3 Institute of Bioscience, Universiti Putra Malaysia, 43400 UPM Serdang, Selangor Malaysia; 4 Department of Clinic and Internal Medicine, College of Veterinary Medicine, University of Sulaimani, Sulaimani City, Kurdistan Region Iraq; 5 UKM Medical Centre, UKM Medical Molecular Biology Institute (UMBI), Cheras, Wilayah Persekutuan Malaysia; 6 Faculty of Pharmacy, MAHSA University, Jenjarom, Malaysia; 7 Faculty of Veterinary Medicine, Universiti Putra Malaysia, 43400 UPM Serdang, Selangor Malaysia

## Abstract

Very recently, we postulated that the incorporation of citral into nanostructured lipid carrier (NLC-Citral) improves solubility and delivery of the citral without toxic effects *in vivo*. Thus, the objective of this study is to evaluate anti-cancer effects of NLC-Citral in MDA MB-231 cells *in vitro* through the Annexin V, cell cycle, JC-1 and fluorometric assays. Additionally, this study is aimed to effects of NLC-Citral in reducing the tumor weight and size in 4T1 induced murine breast cancer model. Results showed that NLC-Citral induced apoptosis and G2/M arrest in MDA MB-231 cells. Furthermore, a prominent anti-metastatic ability of NLC-Citral was demonstrated *in vitro* using scratch, migration and invasion assays. A significant reduction of migrated and invaded cells was observed in the NLC-Citral treated MDA MB-231 cells. To further evaluate the apoptotic and anti-metastatic mechanism of NLC-Citral at the molecular level, microarray-based gene expression and proteomic profiling were conducted. Based on the result obtained, NLC-Citral was found to regulate several important signaling pathways related to cancer development such as apoptosis, cell cycle, and metastasis signaling pathways. Additionally, gene expression analysis was validated through the targeted RNA sequencing and real-time polymerase chain reaction. In conclusion, the NLC-Citral inhibited the proliferation of breast cancer cells *in vitro*, majorly through the induction of apoptosis, anti-metastasis, anti-angiogenesis potentials, and reducing the tumor weight and size without altering the therapeutic effects of citral.

## Introduction

Cancer has become the most dreadful disease where World Health Organization (WHO) has recorded cancer as one of the leading causes of death in the world. Breast cancer particularly is the most common invasive cancer among women worldwide that accounts for nearly one in 3 cancers attacking women in the United States^1^. Various studies have been conducted in finding breast cancer treatment yet there is no ultimate solution. Regardless of the advancement in chemotherapy drugs that are used to treat the breast cancer, the unmanageable side effects caused by it are an unresolved problem. This poor side effect profile of cytotoxic pharmaceuticals has considerably diminished the therapeutic worth of the drug.

Among various plant-based natural product, citral (*Cymbopogon citratus*)has attracted great attention for its flavor and unique properties. Citral is one of the most abundant compounds extracted from the citrus based plants such as lemon and lemongrass. It is a monoterpene aldehyde consisting of isomers geranial and neral combination^2^. The power of citral in combating cancer problem has attracted researchers to improve its efficacy as poor water solubility of citral has limited its anti-cancer therapeutic application. The oral route is a convenient route for the delivery of drugs. However, most of the therapeutic drugs have extremely low levels of oral bioavailability, water solubility, and sustainability problems^3^. In statistic, almost 70% of new drugs formulated are showing poor water solubility, which becomes the limiting factor in the absorption drug after oral admission^4^. Many approaches have been adopted to increase the drug solubility, sustainability, bioavailability and gastrointestinal permeability^5^. Nano-carrier has gained tremendous attention in the development of new pharmaceutical carrier and delivery system. One of the strategies to thwart this problem is to encapsulate citral into the biodegradable and biocompatible nanoparticle.

Nanostructured lipid carrier (NLC) is one of the most recent lipid-based nanoparticles that enhanced the water solubility of the hydrophobic drug, increase the drug loading capacity and stability to accommodate more drug and avoid expulsion. Thus, drug delivery system particularly NLC is engaged to minimize the side effects while maintaining the effectiveness of the drug through prolonged sustainability^6,7^. The physicochemical properties of NLC were confirmed to be suitable as a potential delivery system of citral and nontoxic towards the healthy cell. Currently, there is no study that has been investigated on the NLC-Citral and its therapeutic properties although citral is known to have anticancer properties. To the best of our knowledge, this is the first report on the anti-cancer effects of NLC-Citral on human breast cancer cell line MDA MB-231 and murine breast cancer model.

## Results

### NLC-Citral inhibited the proliferation of MDA MB-231 cells

**Figure 1:**
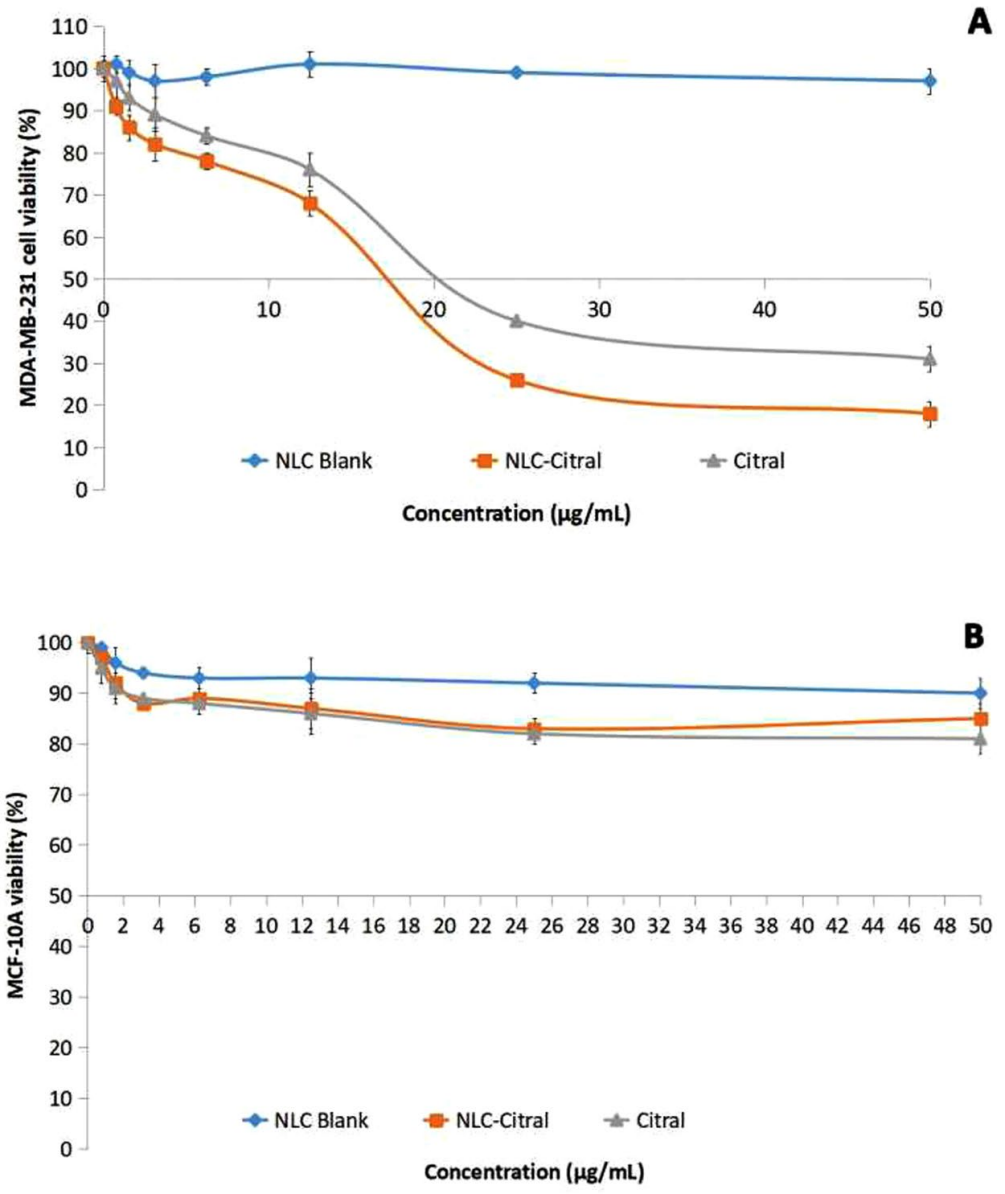
The average value of IC_50_ in MDA MB-231 (**A**) and MCF-10A (**B**) treated with NLC-Blank, NLC-Citral, and citral for 48 hours.

Anti-proliferative effects of NLC-Citral on human breast cancer cell line (MDA MB-231) and the non-transformed mammary epithelial cell line (MCF-10A) were assessed by MTT assay. Cells were treated with 2-fold serial dilutions of NLC-Blank, NLC-Citral, and citral for up to 72 hours. The IC_50_ of NLC-Citral (18.2 ± 0.71 µg/mL) on MDA MB-231 was substantially lower than citral alone (20.5 ± 1.41 µg/mL) (Fig. 1B) at 48 hours treatment. In addition, it can be perceived that the IC_50_ of each treatment on MCF-10A was relatively not detected even at the highest concentration tested (Fig. 1B). Meanwhile, the cytotoxicity of NLC-Blank was concurrently determined using MTT assay. There was no IC_50_ detected for NLC-Blank on both cell lines at 48 hours post-treatment.

Based on the result from MTT assay, 3 doses of NLC-Citral (IC_25_, IC_50_, and IC_75_) (Table 1) on MDA-MB-231 were selected and used for the following assays.

**Table 1:** Values of IC_25_, IC_50_, and IC_75_ of NLC-Citral in MDA MB-231 treated cells for 48 hours.

NLC-Citral (µg/mL)	
IC_25_	10.50 ± 0.90
IC_50_	18.20 ± 0.71
IC_75_	26.80 ± 1.03

### NLC-Citral induced apoptosis effects on MDA MB-231 cells

**Figure 2:**
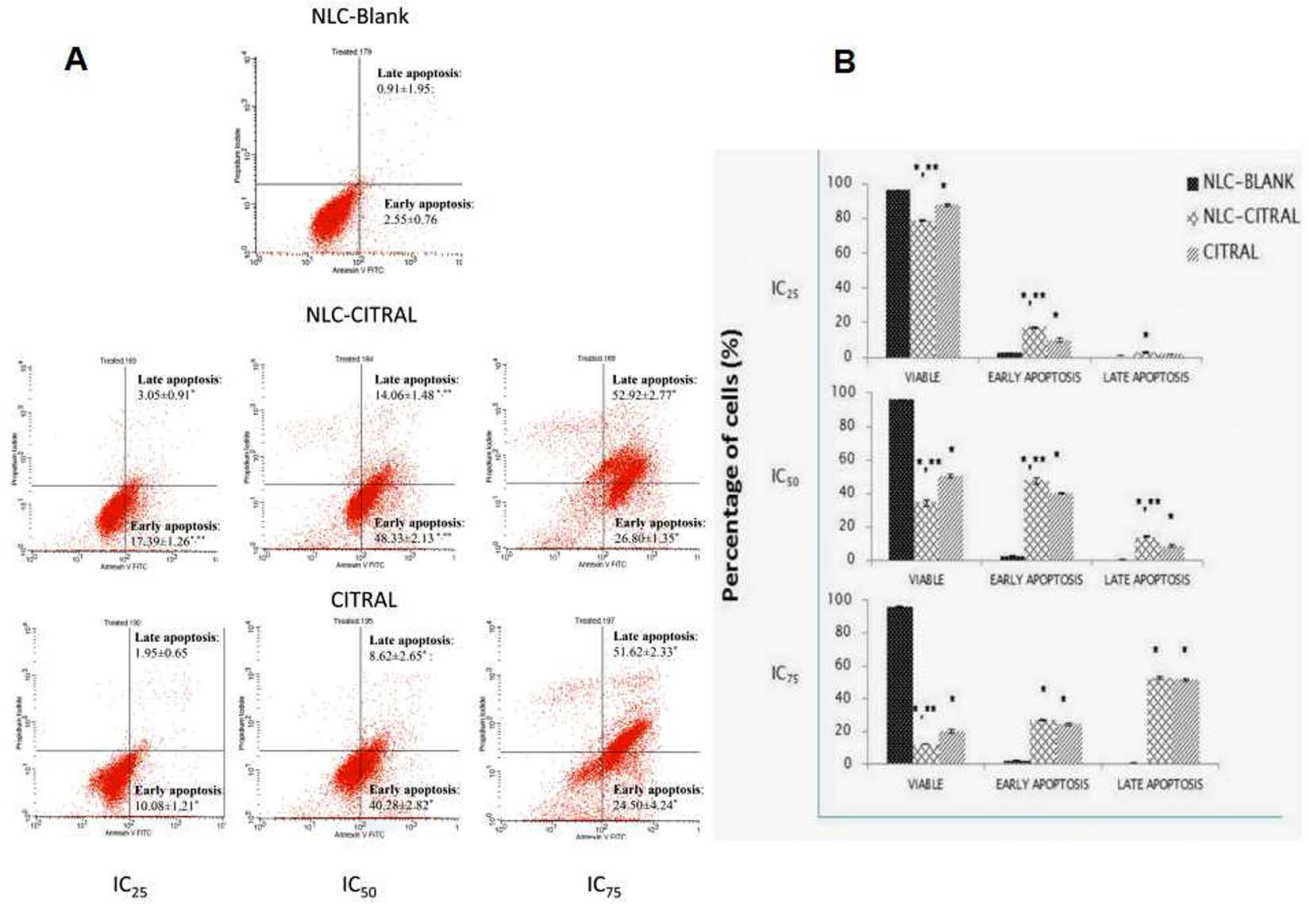
(**A**) Representative histogram analysis and (**B**) Bar chart analysis of the Annexin/FITC assay in MDA MB-231 treated with 3 different doses of NLC-Blank, NLC-Citral and Citral for 48 hours. The experiment was done in triplicate and the data are expressed as mean ± SD. Significance was set at p < 0.05 comparing between groups with (*) to NLC-Blank and (**) to citral.

Annexin V assay was employed to study the apoptosis effects of NLC-Citral and citral on MDA MB-231 cells after 48 hours of treatment by detecting the externalization of phosphatidylserine (PS) to the outer plasma membrane. A shifting pattern in the percentage of viable cells to early apoptosis and to late apoptosis as the concentration increased in both NLC-Citral and citral treated cells can be observed in Fig. 2A. It was observed that the early apoptosis population was significantly (p < 0.05) increased form IC_25_ (17.4 ± 0.8%) to IC_50_ (48.2 ± 1.2%) for NLC-Citral treated cells. However, at IC_75_ most of the cells were shifted from early apoptosis to late apoptosis (53.01 ± 1.0%) for NLC-Citral treated cells (Fig. 2B). A similar pattern was monitored in citral treated cells. This result showed that the apoptosis effects of NLC-Citral and citral toward MDA MB-231 cells were in a dose-dependent manner.

### NLC-Citral promoted the cell cycle arrest at the G2/M phase of MDA MB-231 cells

**Figure 3:**
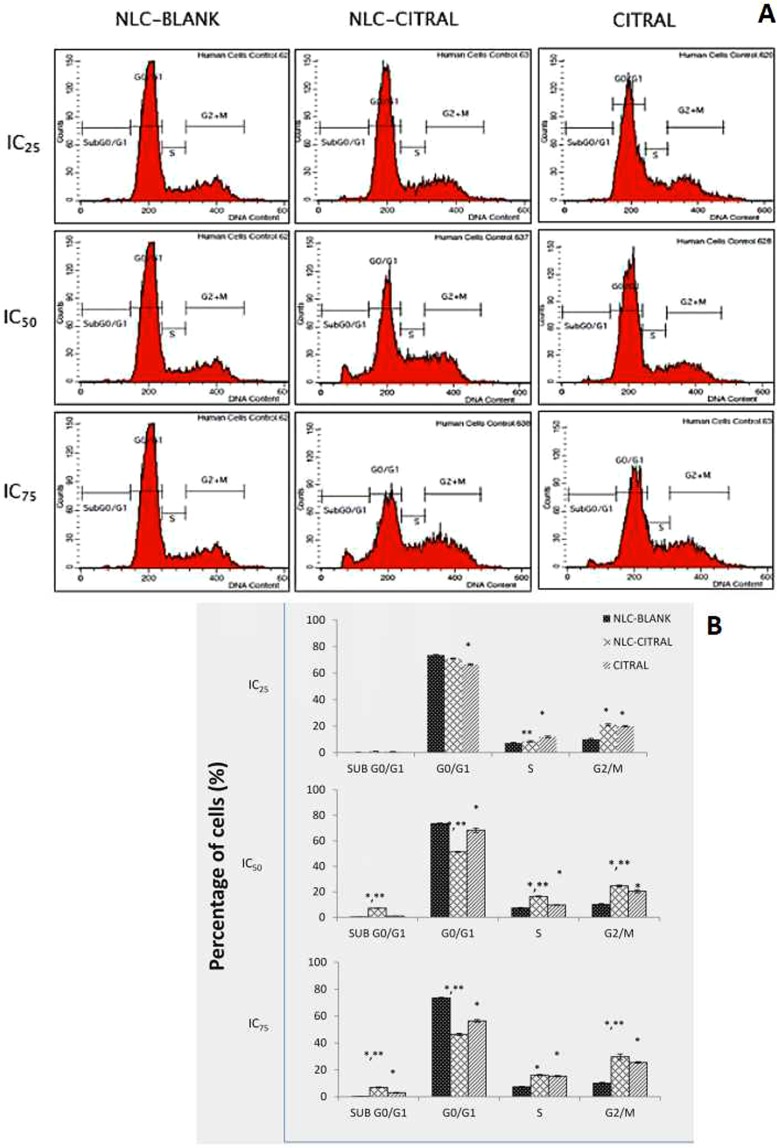
(**A**) Representative histogram analysis and (**B**) Bar chart analysis of the cell cycle in MDA MB-231 after 48 hours of exposure with 3 different concentrations of NLC-Blank, NLC-Citral, and citral. The experiment was done in triplicates and data are expressed as mean ± SD. Significance was set at P < 0.05 comparing between groups with (*) to NLC-Blank and (**) to citral.

MDA MB-231 cells were incubated with 3 different concentrations stated in Table 2 and the cell cycle was examined by flow cytometry. The result showed that percentages of cells at G2/M phase were significantly (p < 0.05) increased as the concentrations of NLC-Citral and citral increased. Based on Fig. 3A, the result shows that NLC-Citral reduced the number of percentage in the G1 phase of the MDA MB-231 cells with concomitant accumulations in G2/M phase. This indicates that there is a G2/M cell cycle arrest in the cells treated with NLC-Citral. The percentage of cells in G2/M escalated to 29.7 ± 0.7% in the IC_75_ NLC-Citral group from the 10.09 ± 0.45% in NLC-Blank at 48 hours post-treatment as depicted in Fig. 3B. As compared to the NLC-Blank, there was a consequential increase in the percentage of cells in G2/M phase as the dose of NLC-Citral and citral elevated from IC_25,_ IC_50,_ to IC_75._ In addition, after 48 hours of treatment with NLC-Citral, this study revealed that the percentage of Sub G0/G1 cells also escalated from 1.21 ± 1.07% in the IC_25_ to 7.21 ± 0.98% in IC_75._

**Table 2:** The validation of mRNA regulation expression level in targeted RNA sequencing versus microarray.

Truseq		Microarray		
NLC-Citral	Citral	Gene Symbol	NLC-Citral	Citral
**Up-regulated**				
2.20	4.30	BAX	1.54	1.05
2.90	2.00	RIPK2	1.15	−1.38
6.30	3.30	PEA15	2.2	−1.15
13.4	4.60	CDKN1B	2.04	3.87
3.10	1.90	BCL2L11	3.20	NS
1.10	1.00	PTEN	NS	8.53
**Down-regulated**				
−5.26E + 00	−2.22E + 00	FZD8	−1.24	−1.04
−3.57E + 00	−3.57E + 00	RELA	−2.06	−1.12
−2.50E + 00	−2.42E + 00	NFKB1	−2.39	−1.08
−2.17E + 00	−9.09E − 01	CDC34	−1.15	−1.04
−2.08E + 00	−2.94E − 01	BCLAF1	−5.69	−3.59
2.60E − 01	1.20E − 01	TGFB1	−3.56	NS

### NLC-Citral exhibited mitochondrial membrane change in MDA MB-231 cells

**Figure 4:**
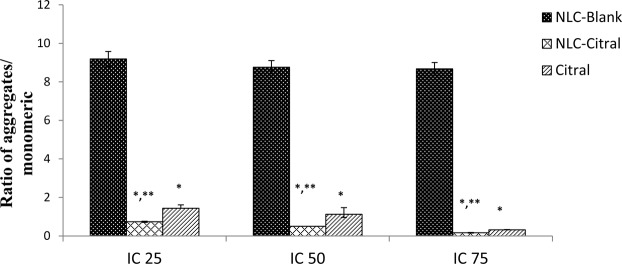
Bar chart analysis of the mitochondrial membrane potential depolarization of MDA MB-231 treated for 48 hours with 3 different concentrations of NLC-Blank, NLC-Citral, and citral. The experiment was done in triplicates and data are expressed as mean ± SD. Significance was set at P < 0.05 comparing between groups with (*) to NLC-Blank and (**) to citral.

Additionally, the changes in the mitochondrial membrane potential of MDA MB-231 cells treated with NLC-Citral and citral were measured with the JC-1 assay. A cell stained with JC-1 dye emits red (aggregates) or green (monomers) fluorescent depending on the accumulation of the JC-1 dye in the mitochondria. In non-apoptotic cells, JC-1 exist as aggregates and can be detected as red fluorescent whereas, in the apoptotic cells JC-1 exist in monomeric form and detected as green fluorescent. According to Fig. 4, there was a decrease in the ratio of aggregate to monomer in the NLC-Citral and citral treated cells as the concentration increased. The higher the dose of the NLC-Citral treatment, the lower the ratio of aggregates to monomers observed. Following treatment, the ratio of aggregates to monomers has significantly deescalated for 0.2-fold from 0.5-fold for NLC-Citral and to 0.3-fold from 1.2-fold for citral as the dose of NLC-Citral escalated to IC_75_ from IC_50_ respectively. The results clearly showed that the induction of apoptosis by NLC-Citral and citral was in a dose-dependent manner. In this study, it was observed that NLC-Blank of all 3 concentrations showed the high ratio of aggregates to the monomer which indicates high mitochondrial membrane potential.

### NLC-Citral activated Caspase 8 and 9 in MDA MB-231 cells

**Figure 5:**
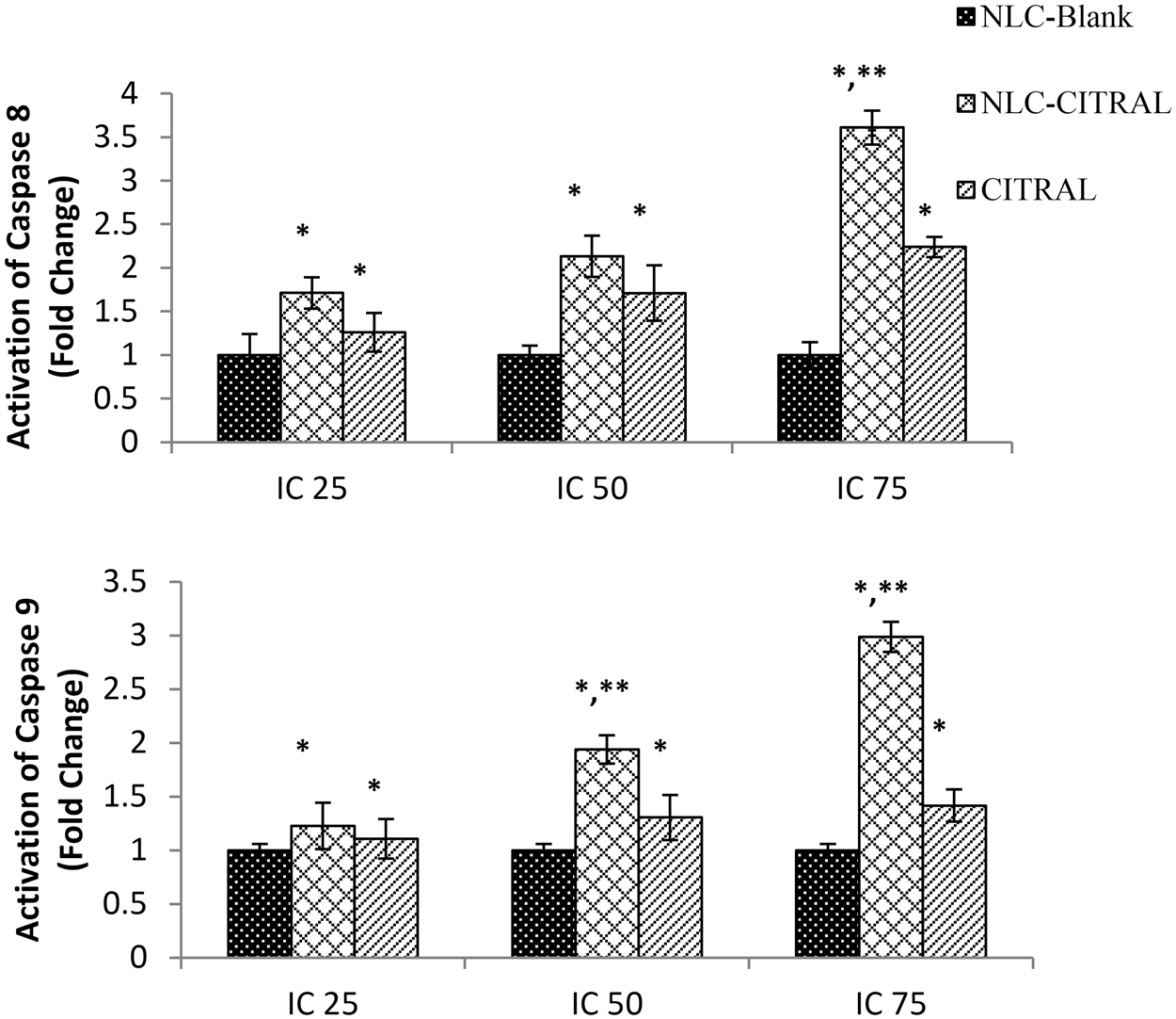
Bar chart analysis of the activation of Caspase 8 and 9 in MDA MB-231 treated with 3 different concentrations of NLC-Blank, NLC-Citral, and citral. The experiment was done in triplicates and data are expressed as mean ± SD. Significance was set at P < 0.05 comparing between groups with (*) to NLC-Blank and (**) to citral.

To further confirm the mode of cell death induced by NLC-Citral, the activation of Caspase 8 and 9 was determined in MDA MB-231 cells using fluorometer. The cells were treated with IC_25_, IC_50_, and IC_75_ of NLC-Citral, citral, and NLC-Blank for 48 hours. The activation of both Caspases was depicted in fold change as compared to the NLC-Blank treated group. Based on Fig. 5, the activation of both Caspase 8 and 9 in MDA MB-231 cell was relatively increased as the concentration of NLC-Citral and citral increase. It can be observed that there was a significant increase in the activation of Caspase 8 by 3.6-fold in IC_75_ from 2.1-fold in IC_50._ Similarly, NLC-Citral has significantly increased the activities of Caspase 9 in the treated cells by 1-fold in IC_75_ from IC_50_. The similar pattern can be seen in the citral treated group. Therefore, the level of Caspase 8 and 9 activations on MDA MB-231 cells was in a dose-dependent manner.

### NLC-Citral inhibited the invasiveness of MDA MB-231 cells

**Figure 6:**
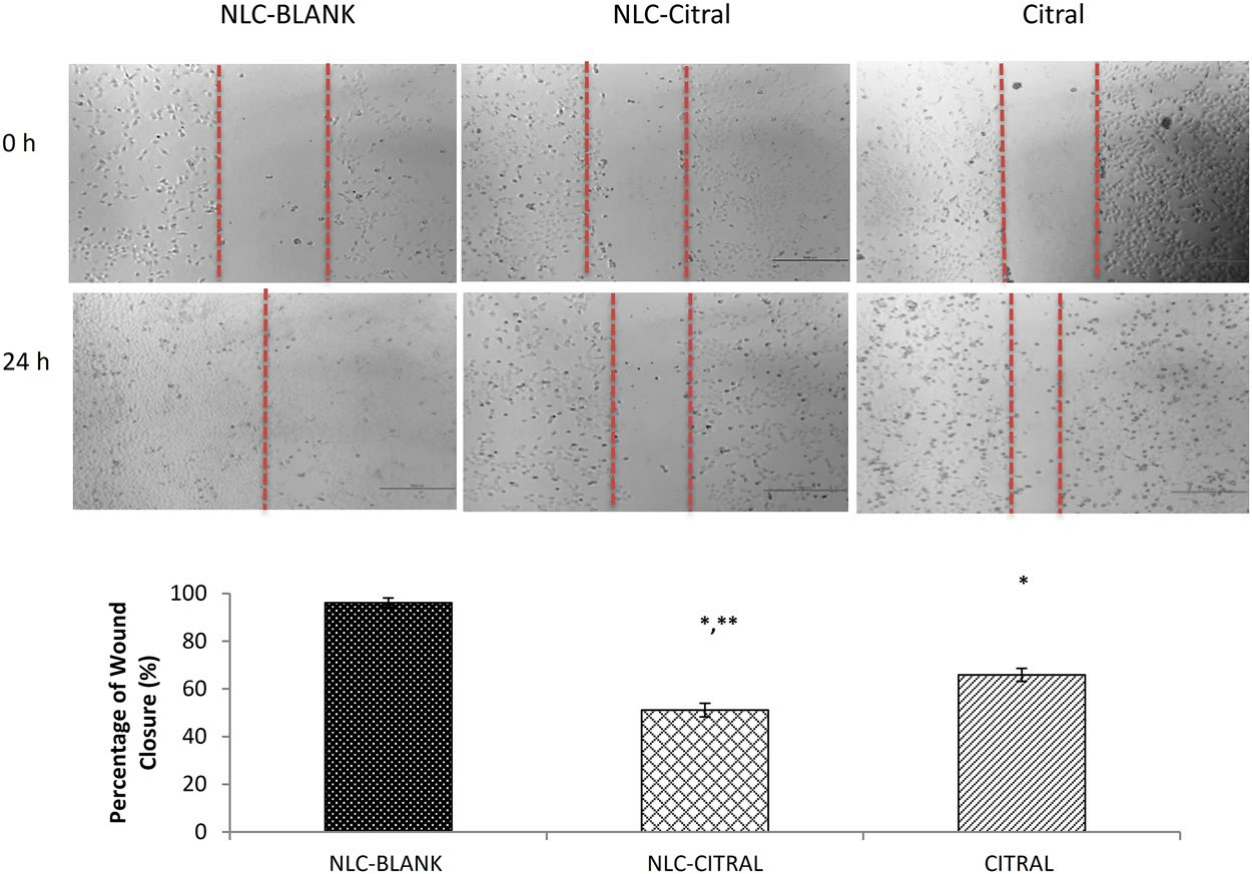
Representative images and bar chart analysis of wound closure in MDA MB-231 cells when treated with 12.5 µg/mL NLC-Blank, NLC-Citral and citral for 24 hours. The size of the wound closure was measured in between the red dotted lines in the pictures. The experiment was done in triplicates and data are expressed as mean ± SD. Significance was set at P < 0.05 comparing between groups with (*) to NLC-Blank and (**) to citral.

Since MDA MB-231 cell line is a highly invasive human breast cancer cells, the wound healing, migration and invasion assays were conducted to study the effectiveness of NLC-Citral as an anti-metastatic agent for human breast cancer *in vitro*. Following treatment with 12.5 µg/mL of NLC-Citral for 24 hours, the substantial decrease in the percentage of wound closure has been displayed. The percentage of wound closure in NLC-Citral treated cells was 51.1 ± 1.22% while it was 95.9 ± 2.05% in the NLC-Blank treated cells (Fig. 6). There was also a significant decrease in the percentage of wound closure in NLC-Citral treated cells as compared to citral treated cells (66.4 ± 2.22%).

**Figure 7:**
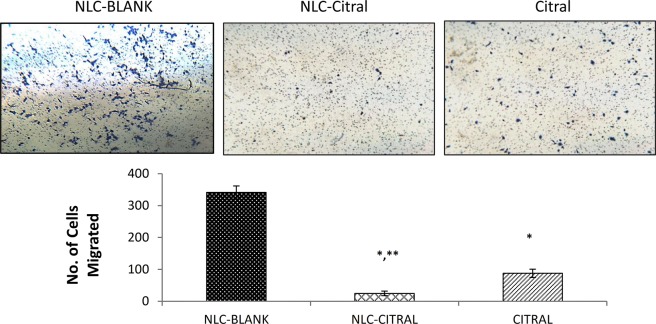
The representative images and bar chart analysis of MDA MB-231 cells upon treatment with 12.5 µg/mL of NLC-Blank, NLC-Citral, and citral. The cells were allowed to migrate through the 0.8 µM pore-sized membrane. The images were viewed at 200X magnification. The experiment was done in triplicates and data are expressed as mean ± SD. Significance was set at p < 0.05 comparing between groups with (*) to NLC-Blank and (**) to citral.

**Figure 8:**
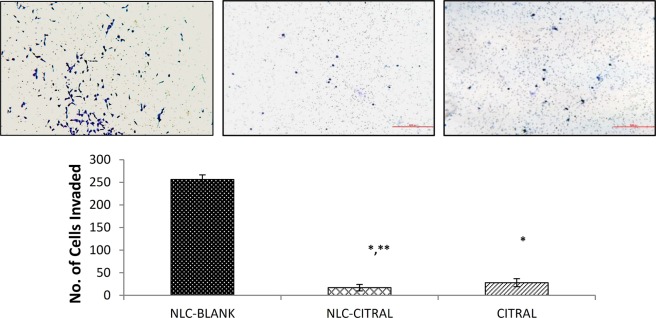
The representative images and bar chart analysis of MDA MB-231 cells upon treatment with 12.5 µg/mL of NLC-Blank, NLC-Citral, and citral. The cells were allowed to invade through the 0.8 µM pore membrane layered with matrigel. The images were viewed at 200X magnification. The experiment was done in triplicates and data are expressed as mean ± SD. Significance was set at p < 0.05 comparing between groups with (*) to NLC-Blank and (**) to citral.

Anti-migration effects of NLC-Citral and citral were further examined through the transwell *in vitro* migration assay. Figure 7 indicates that the number of cells migrated in NLC-Citral was significantly decreased by 13-fold from NLC-Blank. Nonetheless, the citral has declined the number of migrated cells substantially from NLC-Blank by 4-fold. On the other hand, the invasiveness of MDA MB-231 cells was tested under treatment of NLC-Citral and citral alone through a matrigel. This assay was conducted to further study the effectiveness of NLC-Citral in controlling the MDA MB-231 cell’s invasion properties. From Fig. 8, it was clearly shown that the number of invaded cells has decreased significantly in NLC-Citral and citral by 15-fold and 9-fold respectively. Hence, it can be concluded that the NLC-Citral has quenched the migration and invasion abilities of MDA MB-231 cells *in vitro*.

**Figure 9:**
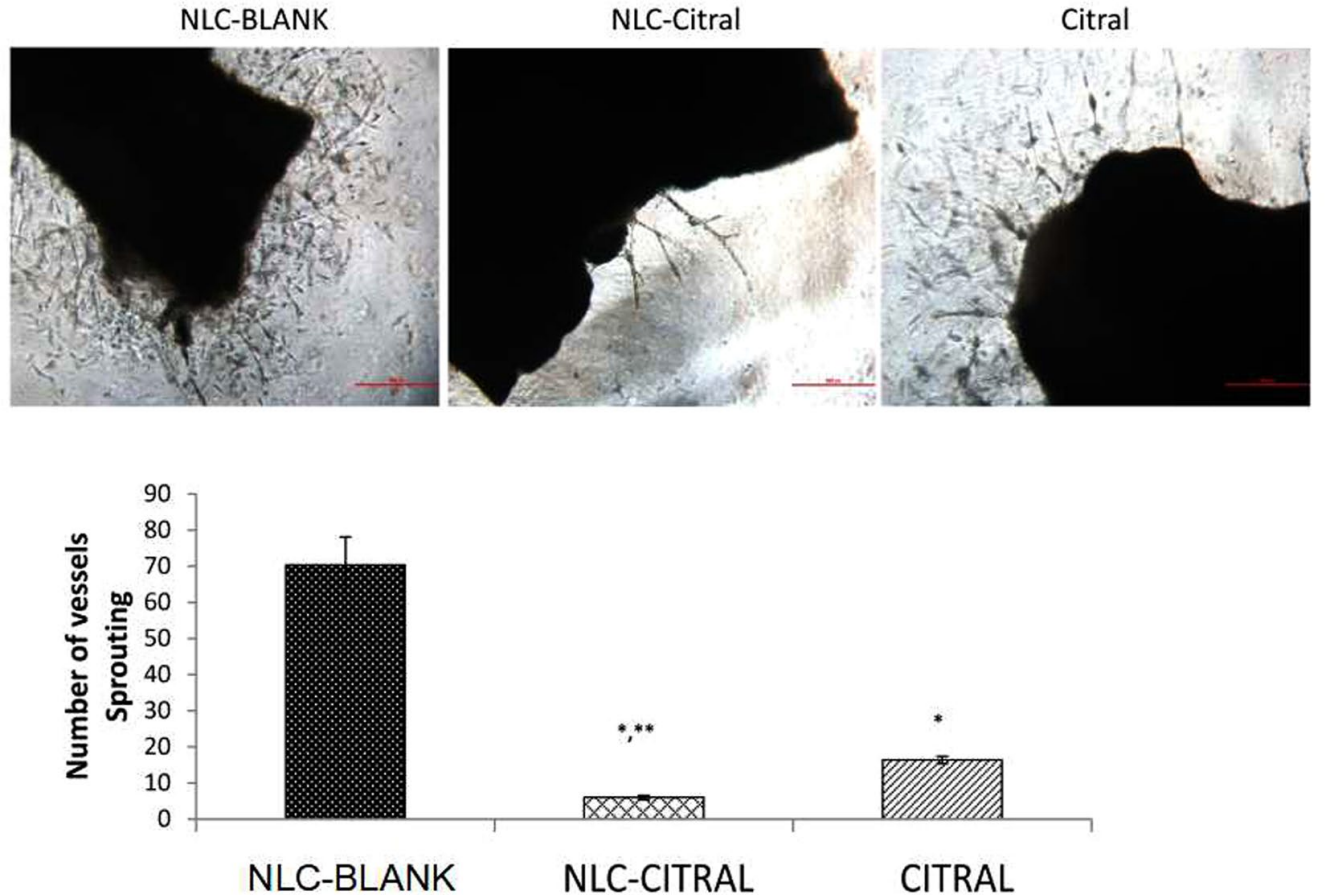
The representative images and bar chart analysis of the *ex-vivo* mouse aorta ring assay when treated with 12.5 µg/mL of NLC-Blank, NLC-Citral, and citral for 24 hours. The presence of the vessels protruding (Red arrow) from the aorta were counted. The experiment was done in triplicates and data are expressed as mean ± SD. Significance was set at p < 0.05 comparing between groups with (*) to NLC-Blank and (**) to citral.

An anti-angiogenic potential of NLC-Citral was investigated using *ex-vivo* mouse aorta ring assay. As depicted in Fig. 9, the number of micro-vessels outgrowth from the thoracic aorta was declined in numbers in an NLC-Citral treated ring as compared to the citral and NLC-Blank. The sprouted vessels formed in the NLC-Citral treated group was reduced by 12-fold as compared to the NLC-Blank group. In contrary, citral treated group showed only 4-foldreduction to NLC-Blank. This implies that the NLC-Citral possessed better anti-angiogenesis potential than citral alone.

### Microarray-based gene expression profiling

About 1100 genes were up-regulated and 1190 were down-regulated from NLC-Citral over control. On the other hand, 1999 genes were up-regulated and 1855 genes were down-regulated in citral versus control, subsequently. Pathway analysis further revealed the molecular processes that associated with the inhibition of cancer cell development of NLC-Citral. In particular, apoptosis, cell cycle mechanism, and metastasis-related pathways were closely examined. These result demonstrated that NLC-Citral and citral were regulated the changes in the expression level of genes in several signaling pathways that are crucial in cancer-associated activities in MDA MB-231 cells when compared to the control group such as the apoptosis, cell cycle mechanism, and metastasis signaling pathways. In brief, it can be observed that Bax gene was highly regulated by 5.48-fold in NLC-Citral while PTEN has increased by 8.53 in citral treated cells. In contrast, in cell cycle pathway CDKN1B was highly regulated (6.87-fold) in citral and PLK-1 has down-regulated to −3.32 fold in NLC-Citral while not significant in citral treated cells. Additionally, the result showed that GJA-1 gene is the most significantly increased gene by 18.32-fold in NLC-Citral with PXDN (−7.53) as the most down-regulated genes in the metastasis-related pathway.

**Figure 10:**
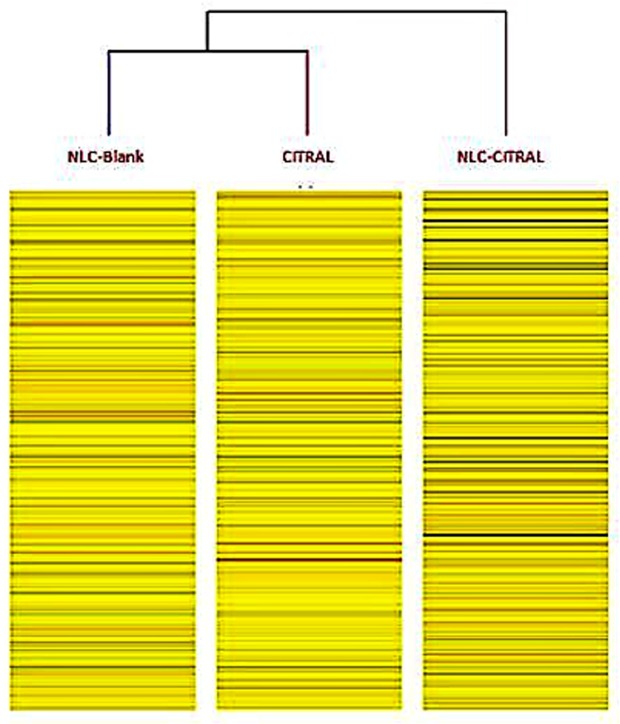
The heat map from microarray cluster analysis after filtering criteria (FC > 2, P > 0.05). Heat map reveals correlations between gene expressions level in different samples. The average differentially expressed genes were analyzed using GeneSpring 13 software for hierarchical clustering based on similarity in between each group.

Cluster analysis provides the better understanding of the degree of association between samples. Based on the heat map displayed in Fig. 10, the differential gene association in NLC-Blank group is closer to citral than NLC-Citral treated group considering the branches formulated in between the group. This showed that the level of gene expression in NLC-Blank is closer to citral than NLC-Citral.

**Figure 11:**
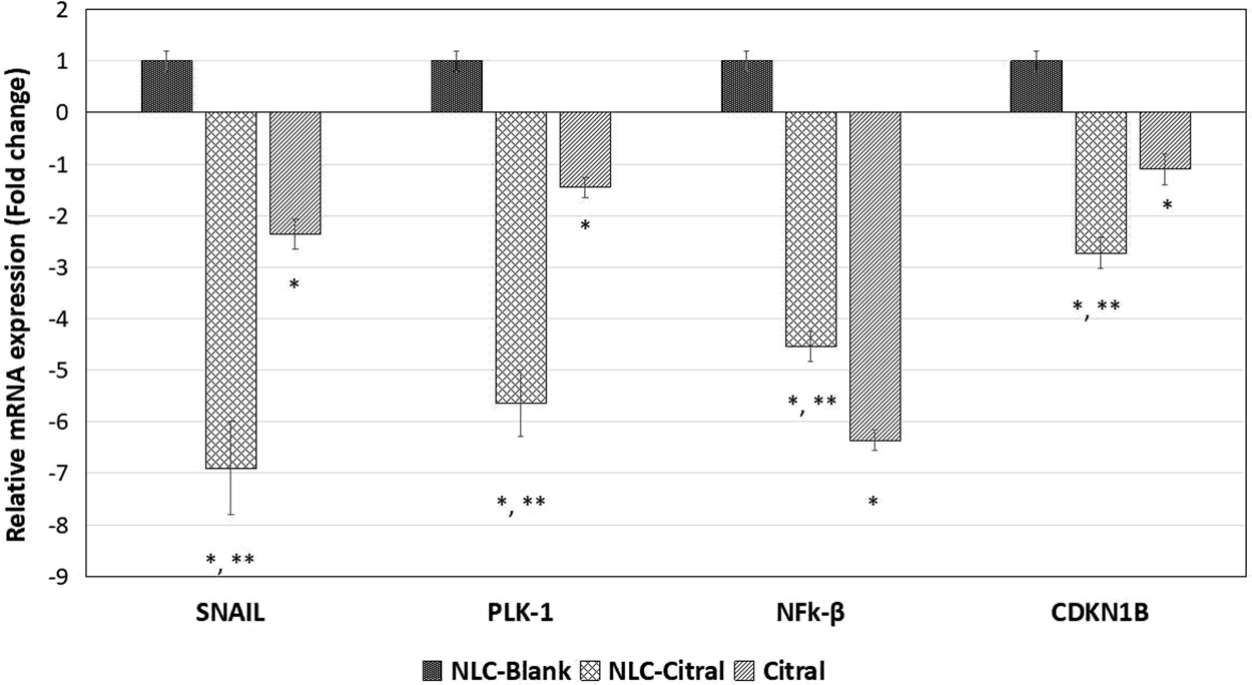
The expression level of mRNA in the qPCR analysis of the MDA MB-231 cells treated with NLC-Citral, citral, and NLC-Blank (Control) on several genes existed in the microarray and Truseq. The experiment was done in triplicates and data are expressed as mean ± SD.

To validate the microarray data, TREX NGS was performed. Seven up-regulated and 5 down-regulated genes were validated. InTruSeq, CDKN11B gene is the most up-regulated genes (13.4-fold) in NLC-Citral treated cells. On the contrary, FZD8 is the most down-regulated gene by 5.3-fold (Table 2). Additionally, 5 genes were selected for additional analysis by qPCR technique. The selected genes were representative of microarray and Truseq data. All genes have expressed similar gene expression pattern that was found in both microarray and TruSeq. More clearly, the expressions of PLK-1, NFK-β and CDKN1B genes have significantly regulated in the NLC-Citral by 5.9, 4.7 and 2.7-fold respectively. Likewise, according to Fig. 11, it is shown that SNAIL gene was down-regulated by 7.3-fold and thus validated the microarray data.

### Proteome profiler analysis

**Figure 12:**
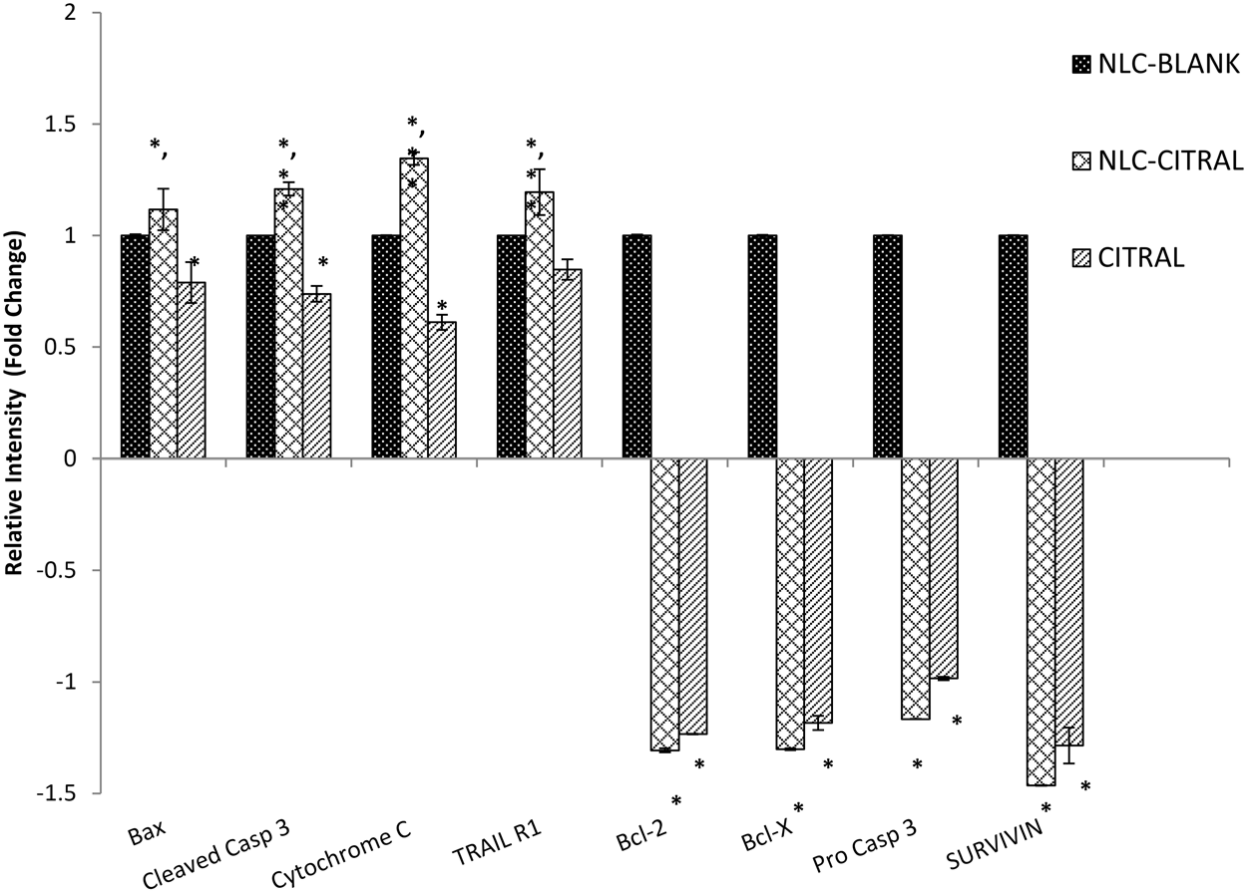
Proteome profiler analysis of the MDA MB-231 cells treated with NLC-Citral, citral, and NLC-Blank (Control) against apoptosis-related proteins treated for 48 hours at IC_50_ concentration. The experiment was done in triplicates and data are expressed as mean ± SD.

Proteome profiler analysis was done to reveal regulation of apoptosis-related proteins by the NLC-Citral and citral treated groups compared to the NLC-Blank. Proteomic analyses of replicates allowed the identification of 8 proteins modulated by NLC-Citral in MDA MB-231. It can be observed that there are 4 identified up-regulated proteins in NLC-Citral treated cells corresponding to Bax (1.2-fold), cleaved Caspase 3 (1.3-fold), Cytochrome C (1.4-fold) and TRAIL R1 (1-fold). On the other hand, 4 down-regulated proteins have been identified which includes Bcl-2 (1.3-fold), Bcl-X (1.4-fold), Pro-Caspase 3 (1.2-fold) and Survivin (1.5-fold) (Fig. 12).

**Figure 13:**
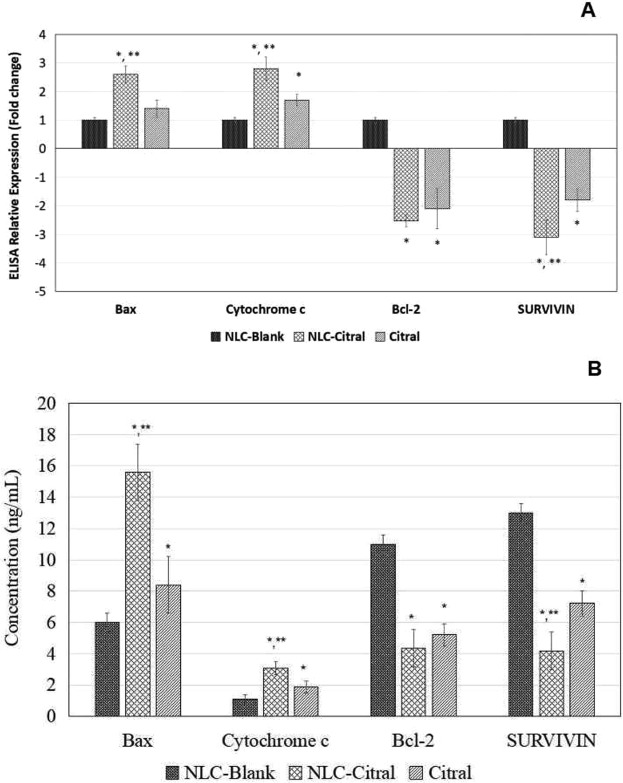
The fold change (**A**) and Concentration (ng/mL) (**B**) levels of protein expression in ELISA test of the MDA MB-231 cells treated with NLC-Citral, NLC-Blank (Control) and citral against apoptosis-related proteins treated for 48 hours at IC_50_ concentration. The experiment was done in triplicates and data are expressed as mean ± SD.

The obtained data from protein profiler was validated using ELISA test in which 2 up-regulated and 2 down-regulated proteins were validated. In ELISA, Cytochrome C protein is the most up-regulated proteins (2.8-fold) in NLC-Citral treated cells. On the contrary, Bcl-2 is the most down-regulated protein by 2.5-fold (Fig. 13).

### NLC-Citral inhibited the proliferation of 4T1 cells *in vitro*

**Figure 14:**
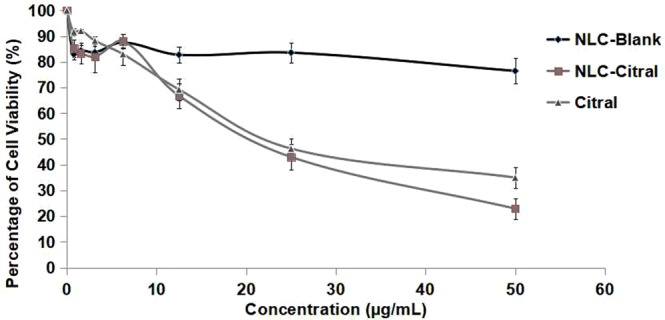
The percentage of 4T1 cells viability treated with different concentrations of NLC-Blank, NLC-Citral, and citral at 48 hours in MTT assay. Each value is represented as mean ± SD and the experiment was done in triplicate.

Based on the MTT assay, NLC-Citral and pure citral have reduced the growth of 4T1 cells *in vitro* in a dose-dependent manner. According to Fig. 14, the half-maximal inhibitory (IC_50_) of NLC-Citral and citral were 20.3 ± 1.76 µg/mL and 21.4 ± 2.82 µg/mL respectively after 48 hours of incubation whilst, for NLC-Blank the cells viability did not reach below 75%.

### NLC-Citral reduced the weight and size of the 4T1 tumor growth in mice

**Figure 15:**
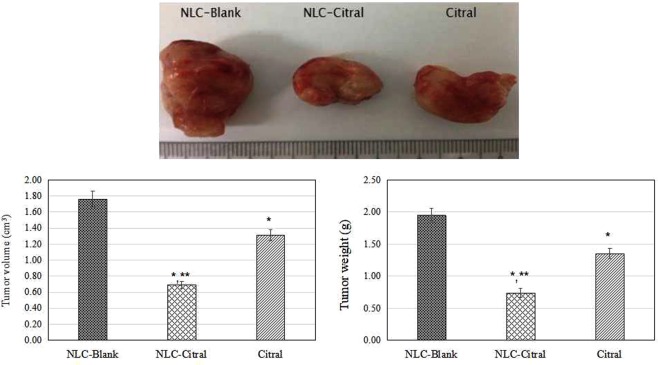
The representative images and bar chart analysis of the tumor weight and size of the mice after 28 days of treatment. The mice were treated with 50 mg/kg/day of NLC-Blank, NLC-Citral and citral via oral delivery. Each value in the bar chart is represented as mean ± SD and the experiment was done in triplicate. Significance was set at p < 0.05 comparing between groups with (*) to NLC-Blank and (**) to citral.

In this study, the weight of the NLC-Citral treated tumor has significantly (p < 0.05) reduced to 0.78 ± 0.04 g as compared to the NLC-Blank (2.0 ± 0.13 g) and pure citral (1.31 ± 0.09 g). At the same time, the size of the NLC-Citral treated tumor also significantly (p < 0.05) reduced to 0.69 cm^3^ as compared to the NLC-Blank (1.76 cm^3^) and pure citral (1.31 cm^3^) (Fig. 15).

## Discussion

From the IC_50_ value of MTT assay, we realized that NLC-Citral inhibited the proliferation of MDA MB-231 better than citral. Nonetheless, there was an insignificant difference in the inhibitory effect of the cancer cells in between the NLC-Citral and citral alone in MDA MB-231 in both time points. Previous studies reported that the incorporation of drug into nanoparticle did not increase the efficiency of the drug in the inhibition of cancer cells^8–10^. In addition, there was no adverse effect on proliferation of normal human breast epithelial cell line MCF-10A at the same dosage and time used for treatments on MDA MB-231 cells. This suggests that the formulation is not toxic to normal cells. Meanwhile, a study suggested that the encapsulation of potential therapeutic agents in lipid nanoparticle generally reduced its toxicity towards normal cells^11^. *In vitro* cytotoxicity test of NLC-Thymoquinone revealed that NLC is safe in normal liver cells (WRL-68)^12^. Furthermore, relatively low toxicity effects from NLC-Blank reported after being tested for 72 hours suggest that the cause of the cell death was clearly not from the NLC as a carrier itself. In addition, it can be observed from the preliminary MTT assay that NLC-Blank demonstrated no cytotoxic effects on MDA MB-231 cells. This suggests that the strong inhibitory effect of NLC-Citral is majorly from the citral. Rationally, the goal of a drug delivery system is to enhance the bioavailability of the drugs towards targeted diseased cells, promoting the required response while minimizing its side-effects^13^. Considering the slow release effects of the citral loaded in NLC to act upon target cells, it is possible that NLC-Citral to be more effective than pure citral in terms of prolonged anticancer activity.

In the present study, it was found that NLC-Citral significantly inhibited the proliferation of breast cancer cell lines via apoptosis. Based on Annexin V assay, there was a shift in the pattern of the externalization of phosphatidylserine from viable to early apoptosis and to late apoptosis as the concentration of NLC-Citral increased. It was reported that citral has induced the apoptosis of MCF-7 breast cancer cells by increasing the percentage of early apoptotic cells^14^. Simultaneously, NLC-Citral was found to trigger the depolarization of mitochondrial membrane potential in MDA MB-231 cells using JC-1 analysis. The depolarization of mitochondrial membrane potential could be linked with the induction of apoptosis in breast cancer cells^15^. Based on the observation, the depolarization of membrane potential in NLC-Citral treated cells was significantly (p < 0.05) higher than in citral treated cells which reflects apoptotic cells.

Microarray expression profiling and protein assay analysis was performed to gain insight into the molecular activities and pathways related to apoptosis effects of NLC-Citral and citral in MDA MB-231 cells and finally, the results were validated using ELISA technique. Numbers of study have been done in the discovery of biomarkers involved in breast cancer in the identification of genes related to metastasis of breast cancer to other organs^16,17^. Therefore, in this present study, the regulation of differential gene expression that associated with apoptosis, cell cycle mechanism, and metastasis of MDA MB-231 cells treated with NLC-Citral and pure citral with NLC-Blank as a control were determined.

Microarray analysis showed that the induction of apoptosis was associated with the up-regulation of Bax and Bcl-2L11 genes level in MDA MB-231 cells. Generally, Bax is regulated by the other members of the Bcl-2 family to form oligomers and puncture the mitochondrial outer membrane to mediate cell death by apoptosis^18^. Copper oxide nanoparticle (CuO-NP) was confirmed to induce apoptosis in K562 cells through mitochondria-mediated pathway as evidenced by the up-regulation of Bax genes in the treated cells^19^. On the other hand, Bcl-2L11 is a well-known gene to mediate cell apoptosis when it is induced by several apoptotic stimuli such as cytokines, radiation and cytotoxic peptide^20^. Recently, it was reported that the association of Bcl-2L11 gene with miR-24 holds potential targets for gene therapy of gastric cancer cells^21^. Interestingly, both of these genes were highly regulated in the NLC-Citral treated cells as compared to the pure citral. This suggests that the regulation of pro-apoptotic genes among the Bcl-2 family was induced better by the NLC-Citral.

In addition, PTEN gene is another gene that was highly up-regulated in an apoptosis-related pathway. Phosphatase and tensin homolog deleted from chromosome 10 (PTEN) is a tumor suppressor that can inhibit cellular proliferation, survival, and growth by inactivating PI3-kinase-dependent signaling pathway^22^. Over-expression of PTEN in cancer cells results in cell cycle arrest, cell death, and metastasis. The study reported that PTEN has therapeutic potential for ovarian cancer through the inhibition of angiogenesis in mice tested^23^. Additionally, it has been documented that PTEN could enhance the sensitivity of anticancer agents in ovarian cancer and in human bladder cancer cells^24,25^. Intriguingly, in this case, pure citral has significantly induced the up-regulation of PTEN in MDA MB-231 treated cells than NLC-Citral. This implies that NLC-Citral and citral may regulate differently in different types of a gene in the induction of apoptosis signaling pathway. The previous study has demonstrated that the level of hTERT and PinX genes expression were different in Calu-6 cells treated with nanoparticle loaded curcumin and pure curcumin at the same concentrations and conditions^26^.

Subsequently, proteome profiler analysis proved that NLC-Citral and citral increased the protein expression of Bax, cleaved Caspase 3, TRAIL R1 and Cytochrome C in MDA MB-231 cells while decreased the Pro-Caspase 3, Bcl-2, Bcl-X and Survivin proteins. The Bcl-2 family is consist of a number of protein that plays a crucial role in the regulation of apoptosis where some members of the Bcl-2 family such as Bax appear to induce apoptosis while the other like Bcl-2 and Bcl-X can inhibit apoptosis^27^. Bax is a pro-apoptotic protein that resides in the outer mitochondrial membrane^28^. The up-regulation of Bax triggered a release of Cytochrome C from mitochondria and induced caspases activation into the cytosols^29^. Several studies had suggested that Bax could reduce the mitochondrial membrane potential by creating pores in the membrane for Cytochrome C to escape and eventually cell death^30,31^. Moreover, the release of Cytochrome C was upstream of the initiation of Caspase cascade including Caspase 8, 9 and 3 in enhanced apoptosis^32^. The activation of Caspase 9 may amplify the mitochondrial disruption for the efficient apoptosis executor during intrinsic apoptosis^33^. Besides that, Caspase 8 is involved in the extrinsic pathway of apoptosis and usually recruited by the ligand binding of death receptors and tumor necrosis factor^34^. The activation of death receptor ligand, TRAIL R1 lead to the recruitment of Caspase 8 which triggers a Caspase cascade and subsequently induced apoptosis of susceptible cells^35^. Meanwhile, the induction of cleaved Caspase3 protein expression and the inhibition of pro Caspase 3 in the cells directly targeted the mitochondria for cell death to occur^36^. In the previous study, citral has been found to be a potent inducer in the activation of Caspase 3 enzymatic activity in tumor cell lines^37^. Interestingly, the NLC-Citral was seen to significantly stimulated Caspase 9 and Caspase 8 activities in a dose-dependent manner. Therefore, this result suggested that the apoptogenic effect of NLC-Citral on MDA MB-231 cells is via both the extrinsic and intrinsic pathways.

Furthermore, Bcl-2 and Bcl-X are anti-apoptotic proteins that highly expressed in the chronic cancers. Over-expression of Bcl-2 and Bcl-X prevent the Cytochrome C to initiate the apoptosis by blocking it to be released from the mitochondria thus the apoptosis mechanism to take action^38,39^. Survivin protein was observed to be down-regulated in the NLC-Citral and citral treated cells. Survivin was demonstrated to inhibit the apoptosis by interfering with the function of Caspase 3, 7 and 9 in apoptosis-related pathway either directly or in indirect ways^40^. The study had revealed that high expression of Survivin was associated with the inhibition of apoptosis in 167 of breast cancer patients^41^. In cancer cells, the induction of Survivin is related to the increase in cells proliferation, inhibition of apoptosis, and resistance to chemotherapy^42^. Hence, the regulation of proteins and genes studies represents an important molecular mechanism by which NLC-Citral induced breast cancer apoptosis.

Cellular mechanism studies showed that NLC-Citral affected the MDA MB-231 cell cycle machinery. One of the factors in antiproliferative activity of NLC-Citral on MDA MB-231 is the target of cell division by arresting cells at the G2/M phase which is analyzed through flow cytometer system. The key part of cell cycle machinery is the cyclin-dependent kinases and the regulatory protein called cyclins. Superficially, the connection between the cell cycle and cancer is obvious as the cell cycle machinery controls cells proliferation. Several therapeutic agents include flavopiridol, AZD5438, bryostatin-1 and indisulam are targeting the regulation of expression and activity of cyclin-cyclin dependent kinases complexes in cell cycle process^43^. This occurrence was further confirmed with the microarray as the result showed that NLC-Citral and pure citral has regulated the cell cycle signaling pathway. Microarray analysis indicated the down-regulation of PLK-1 gene in NLC-Citral treated cell while this was not significantly detected in citral treated cells. The study revealed that over-expression of PLK-1 contributed to many tumor types, including breast cancer, colorectal cancer, ovarian cancer, non-small lung cancer and many more^44^. The depletion of PLK-1 gene expression was directly promoted G2/M phase arrest in the human cancer cells^45^. In addition, pre-clinical studies using small interfering RNAs (siRNA) inhibited PLK-1 resulting in the G2/M arrest, apoptosis and tumor growth inhibition^44^. As depicted in the microarray analysis, NLC-Citral managed to inhibit the expression of PLK-1 gene in the treated cells. Cell cycle checkpoints help the DNA replication and divisions. Checkpoints are regulated by a family protein kinases, CDKs. Mutation in G2 checkpoint prevents the entry of mitosis in cell cycle process and undergo apoptosis^46^. Cyclin-dependent kinases, on the other hand, are activated by cyclin binding and inhibited by CKD inhibitors^47^. Cyclin B is another gene associated with the cell cycle deregulation in cancer cells. CCNB1 gene was down-regulated by the NLC-Citral and pure citral treatments. CCNB1 is known as Cyclin B1 gene that required for the activation of mitosis factor upon the binding of CDK-1 for mitotic initiation^48^. The study reported that down-regulation of Cyclin B1 promoted G2/M phase arrest in the leukemia cells treated with berberine^49^. Prystowsky *et al*.^50^ studied that the inhibition of Plk1 and Cyclin B1 expressions results in panobinostat induced G2 delay and mitotic defects in the head and neck carcinoma cells. Additionally, CDKN1B gene was regulated in both NLC-Citral and pure citral treated cells. CDKN1B is a cyclin-dependent kinase 1 beta inhibitor and widely known as p27. P27 is a major key player in the cell cycle arrest that prevents the activation of Cyclin E or Cyclin D complexes in the cell cycle machinery^51^. In conclusion, these results have an agreement with previous studies showing that citral has an anti-cancer effect on MCF-7 and NB4 cells via the induction of cell cycle arrest at G2/M phase^52,53^.

Approximately, 90% of cell cancer deaths are related to metastasis, therefore the prevention of cancer cell distribution and secondary tumor formation is a major goal of cancer therapy^54^. Anti-metastasis activity of NLC-Citral on MDA MB-231 cells was elucidated through several assays including wound healing. This method is suitable to study cell-matrix interactions and cell migration activities^55^. The results demonstrated thatNLC-Citral has potent activities in inhibiting migration and invasion of breast cancer cells *in vitro*. Metastatic cascade can be divided into several crucial steps that include migration and invasion of the cancer cells into the circulatory system^56^. From mouse aorta ring assay, the number of microvessels was significantly decreased in NLC-Citral. The previous publication suggests that ascorbate has anti-angiogenesis effects through the decreased number of outgrowths of endothelial tubes from aorta ring treated groups^57^. Further, substantiate the metastasis effect of NLC-Citral on breast cancer cells, the metastasis-related genes expression analysis from microarray was also measured.

Angiogenesis is a critical step in tumor growth. Several chemotherapeutic agents were developed by targeting the angiogenesis machinery in breast cancer cells^58^. From the microarray analysis, GJA1 or also known as Connexin 43 (CX43) was the most highly up-regulated gene in the NLC-Citral treated cells. GJA1 gene is a component of gap junctions that allow the transfer of small molecules between adjacent cells. The study has reported that the regulation of gap junctions was pivotal for quantum dot cytotoxicity effects on human mesenchymal stem cells^59^. In addition, the study was revealed that down-regulation of CX43 gene inhibited an anti-angiogenic gene THRSB1 and increased the VEGF gene in the breast cancer cells^60^. Taken together, this result suggests that NLC-Citral and pure citral managed to induce the anti-angiogenic effects on MDA MB-231 cells. Silver nanoparticle was examined to increase the gap junction intercellular communication (GJIC) in the human lung adenocarcinoma cells, A549 through the significant increase of CX43 expression in the cells^61^. On the contrary, the result of the present study showed that Peroxidasin (PXDN) was the most down-regulated gene in MDA MB-231 treated cells. PXDN was on the list of most frequently mutated genes in the third stage pancreatic cancer patients through the prevalence screened^62^. It is an adhesion molecule involved in the extracellular matrix formation (ECM). PXDN was demonstrated as one of the highly regulated genes in plasma protein changes associated with tumor progression study in human breast cancer cells^63^. Besides that, the PXDN expression was relatively high in a few studies including breast, melanoma, ovarian as well as brain tumor samples and it is absent from the normal tissues^64–66^. In addition, microarray result also showed the down-regulation of SNAIL gene in the treated cells. SNAIL is crucial in the embryonic development and cancer progression. Over-expression of SNAIL enhances the metastasis activity of breast cancer cells through the increase of cell motility and invasiveness^67^. In the late stage of cancer, TGF-β has been studied to regulate the EMT by the induction of SNAIL expression in response to tumor progression^68^. It has also been reported that the over-expression of SNAIL in the prostate cancer cells is associated with the secretion of PXDN gene in the cell’s extracellular matrix^69^. According to the microarray analysis, TGF-βI gene was down-regulated in the treated MDA MB-231 cells. Alteration in TGF-βI may induce the angiogenesis stimulation and immune system suppression by promoting the favorable environment for cancer cells development^70^. The recent study supports the idea that over-expression of TGF-βI gene induced cell migration through the lymphatic vessels^71^. On the other hand, the up-regulation of THSB1 gene was also observed. Studies have demonstrated that the up-regulation of THSB1 is an inhibitor of angiogenesis and tumor growth through the direct effects on cell migration and survival^72^. The result of gene expression analysis has supported the anti-proliferative and anti-metastasis effects that were previously measured through cytotoxicity assays and metastasis assays.

Microarray technology allows us to perform high-throughput screening changes in gene expression level. In this study, targeted RNA sequencing assay (Truseq) and quantitative real-time PCR (qPCR) were employed to validate the gene expression from microarray data. Truseq is capable of examining a wide dynamic range of gene expression in a single experiment from a very small amount of sample, thus beneficial for the validation of gene expression level regulated in microarray^73^. Nevertheless, qPCR was done to double confirm the microarray and Truseq data as Truseq is a new method employed with so little references to refer. In fact, most of the studies were use qPCR to validate a few genes regulated in microarray^74^. All selected genes expressed similar gene expression patterns that were found in microarray analysis. Furthermore, to ascertain the potential of antitumor effect of NLC-Citral, the oral efficacy of NLC-Citral as compared to the citral in the inhibition of breast cancer tumor growth was tested *in vivo* with 28 days of treatment in 4T1 challenged mice. The size and weight of the tumors in the NLC-Citral treated mice were found to be substantially reduced compared to the NLC-Blank and citral groups reflecting that NLC-Citral is more effective compared to citral alone. A study has reported that the formulation of citral into biocompatible PEG micelles has significantly inhibited 4T1 tumor growth compared to the citral and control^75^. The results from this study provide critically important experimental evidence to suggest that NLC-Citral may be a novel efficacious and safe agent to inhibit the growth and metastasis of breast cancer.

In conclusion, this study suggests that NLC-Citral holds a promising cytotoxic activity targeting of breast cancer. Overall, the cytotoxic effect of NLC-Citral occurs via the induction of apoptosis and the effect is in a dose-dependent manner. Loading the citral into NLC system did not inhibit the *in vitro* cytotoxic, anti-proliferation and anti-metastatic effects of citral on breast cancer cells. However, NLC-Citral and citral have shown to regulate the different level of gene expression in MDA MB-231 treated cells. The current study shows that the NLC can be used as a carrier for delivery of citral in the breast cancer therapy for the first time. Hence, NLC-Citral can be developed into an ideal carrier for the delivery of citral in combating breast cancer in cancer therapeutics. Nonetheless, further in depth analysis will be done in our future study for *in vivo* experiments to test this hypothesis and also to investigate further roles of the NLC-Citral as the potential anticancer agent.

## Materials and Methods

All methods and procedures used were carried out in accordance with relevant guidelines and regulations. Additionally, all experimental protocols used were approved by Faculty of Biotechnology and Biomolecular Sciences, Universiti Putra Malaysia. All data are compared to NLC-Blank in order to confirm that the anticancer effects of NLC-Citral are due to the citral and not to the components of NLC^10^.

### Preparation and characterization of NLC-Citral

Citral was loaded into NLC by high-pressure homogenization technique using hydrogenated palm oil (HPO), lipoid S-100 (Merck Millipore, Germany), olive oil (Basso, Italy), thimerosal, D-Sorbitol, Tween-80 and citral 95%. Characterization of NLC-Citral was determined using Transmission Electron Microscope (TEM), zeta potential (ZP), entrapment efficiency (EE), and drug loading capacity (DLC). TEM and zeta sizer analyses revealed that NLC-Citral was a nano-size particle with an average diameter of 54.12 ± 0.30 nm. Meanwhile, ZP, EE, and DLC of NLC-Citral were −12.73 ± 0.34 mV, 98.9 ± 0.124%, and 9.84 ± 0.041% respectively^76^.

### Cell lines

The human breast cancer cell lines MDA MB-231 (ATCC, HTB-26), MCF-10 (ATCC, CRL-10317), and murine breast cancer cell line (4T1) were purchased from the American Type Cell Culture Collection (ATCC, Maryland, USA). MDA MB-231 cells were maintained in Dulbecco’s Modified Eagle Medium (DMEM), whereas MCF-10A cells were maintained in DMEM-F12 medium supplemented with hydrocortisone (0.5 µg/mL), insulin (10 µg/mL), and hEGF (20 ng/mL). On the other hand, the 4T1 cells were maintained in a complete growing media (RPMI-1640) (Sigma-Aldrich, USA). All media were supplemented with 10% of fetal bovine serum (FBS) (PAA, Austria) and 1% of penicillin-streptomycin (P/S) (Gibco, USA)^77^.

### MTT assay

MTT assay was conducted in accordance with the previous study with slight modifications^78^. Briefly, MDA MB-231, MCF-10A, and 4T1 cells were harvested, counted and seeded at 1 × 10^4^ cells per well in the 96-well plate for 24 hours. The following day, cells were treated with various concentrations of the sample and incubated for 48 and 72 hours at 37 °C with 5% CO_2_. After that, 20 µL of 5 mg/mL MTT (Merck, USA) reagent was added to each well and incubated for 3 hours. Next, the solution was removed and 100 µL of dimethyl sulfoxide (DMSO) was added to the wells. Then, the plate was read at 575 nm by using the microtiter plate reader (µQuant, Bio-Tek Instrument). The results were analyzed as the percentage proliferation of thecells in respect to the concentration of the samples treated.

### Annexin V fluorescence assay

The apoptosis effect of MDA MB-231 cells was studied using an Annexin V fluorescence Kit (BD Pharmingen, USA) according to manufacturer’s instruction without any modifications. Briefly, cells were seeded for 24 hours at a density of 1.8 × 10^5^ cells per well in 6-well plate. Then, the seeded cells were treated with the designated concentration (Table 1). At 48 hours post-treatment, the treated cells were harvested and collected as a pellet. Next, the pellet was resuspended in 400 µL binding buffer and stained with 5.0 µL of FITC-AnnexinV and 5.0 µL of PI provided in the kit. Afterward, the cells were analyzed by BD FACS Calibur (Becton Dickinson, USA). The result was analyzed using CellQuest 3.3 software. The experiment was performed in triplicates^79^.

### Cell cycle analysis by flow cytometer

The effect of NLC-Citral on the cell cycle of MDA MB-231 cells was investigated using flow cytometry cell cycle analysis. Cell cycle assay was carried out using the CycleTEST Plus DNA Reagent Kit according to the user’s guideline (BD Pharmingen, USA). Firstly, cells were seeded at a density of 1.8 × 10^5^ cells/well for 24 hours. Then, cells were treated with 3 different dosages of NLC-Citral based on the result of MTT assay (Table 1). After 48 hours post-treatment, cells were collected and the pellet was resuspended in 200 µL of trypsin (solution A) and RNase (solution B) for 10 minutes each prior to 250 µL of solution C (propidium iodide (PI)) for 15 minutes. After that, flow cytometric analysis was performed using BD FACS Calibur (Becton Dickinson, USA) within 3 hours. The result was analyzed using CellQuest 3.3 software. The experiment was performed in triplicates^80^.

### JC-1 mitoscreen assay

JC-1 assay was used to detect the depolarization of the mitochondrial membrane potential of the cell after being treated with the selected doses of NLC-Citral for 48 hours using BD MitoScreen Kit (BD Pharmingen, USA). In brief, the cells were seeded in 6 wells plate for 24 hours at a density of 1.8 × 10^5^ cells per well. Then, the cells were treated with various concentrations described in Table 1. This assay was performed based on the manufacturer’s instruction provided with the kit without any modifications. After 48 hours of incubation, the cells were harvested, collected, and incubated with 500 µL of JC-1 stock solution for 15 minutes. Then, it was preceded for analysis by BD FACS Calibur (Becton Dickinson, USA). The result was analyzed using CellQuest 3.3 software. The experiment was performed in triplicates^81^.

### Caspase 8 and 9 fluorometric detection assay

The activity of Caspase 8/9 in MDA MB-231 cells treated with selected doses of NLC-Citral for 48 hours was examined. This activity was determined using CaspGLOW Red Active Caspase 8 and 9 Staining Kit (BioVision Inc, USA). Cells were seeded in 6 wells plate at a density of 1.8 × 10^5^ cells per well. The next day, cells were treated at desired concentrations as described in Table 1. Then, cells were harvested at 48 hours post-treatment and the pellet was stained with 1.0 µL of RED-IETD-FMK for Caspase 8 and 1.0 µL of RED-LEHD-FMK for Caspase 9. The stained pellet was then incubated in the 37 °C incubator for 30 minutes. Finally, the cells were washed twice with PBS prior to being analyzed using Fluorometer (Thermo Fisher Scientific, USA). The procedure was followed according to the manufacturer’s instructions without any modifications.

### *In vitro* wound healing assay

This assay was performed according to the protocol defined in a previous study with slight modifications^82^. MDA MB-231 cells were plated to create 80% confluent monolayer overnight. After 24 hours incubation, the cell monolayer in a straight line scraped with a sterile p200 pipette tip to create scratches. Subsequently, the media was replaced with the treated media. Cells were then incubated for 24 hours. The image of wound closure was captured at 0, 12 and 24 hours of incubation time (Nikon, Japan).

### *In vitro* migration and invasion assay

The *in vitro* migration and invasion assays were done to see the ability of the cells to migrate and invade through the trans-well membrane^83^. Prior to the experiment, the cells were serum-starved by replacing the DMEM with serum-free DMEM for 24 hours. This was done based on the predicament that MDA MB-231 cells are able to migrate and invade through the membrane by the attraction of attractants. For invasion assay, the cell culture insert membrane (SPL, Korea) was coated with 650 µL of diluted matrigel and incubated for 3 hours. Regarding, migration assay, the cells at a density of 4 × 10^5^ cells per well were seeded on top of the solidified membrane. Meanwhile, in the bottom compartment of the chamber, 2.0 mL of supplemented media with 15 µg/mL of NLC-Citral were added. Then, the cells were incubated for 24 hours at the incubator. After that, the non-migrated/invaded cells at the top part of the membrane were scraped away with the cotton swab. The lower part of the membranes was fixed with 1.0 mL methanol for 30 minutes and then preceded with 0.5% crystal violet staining for another 30 minutes. The cells were counted from various fields and sets of the membrane at an area of 27 cm × 20 cm and this assay were performed in triplicates. Untreated MDA-MB-231 cells were used as the positive control to confirm the cell migration in the experiment.

### *Ex vivo* mouse aorta ring assay

The aorta ring assay provides a better understanding of angiogenesis process, in which it allows the analysis of developing microvessel branching and migration over the timescale^84^. Thoracic aorta was removed from the 7 weeks old BALB/c mice and immediately transferred to a petri-dish containing sterile PBS. The aorta ring was rinsed thrice before being cut into 1.0 mm long. The sectioned aorta was then transferred into 48-well plate pre-coated with 100 µL of matrigel (BD, USA). Next, another layer of matrigel was placed on top of the aorta subsequently. Once the gel was solidified, Opti-MEM media (Life Technologies, USA) with the desired concentration of NLC-Citral was added into the well. The plate was incubated at 37 °C with 5% CO_2_ for 7 days and viewed under an inverted microscope (Nikon, Japan). The aorta was photographed and analyzed based on the number of protruding vessels from the aorta. This study was performed in accordance with relevant approval by the Animal Care and Use Committee (ACUC), Faculty of Veterinary Medicine, Universiti Putra Malaysia (UPM/IACUC/AUP-R098/2014).

## Gene Expression Profiling

### RNA extraction

MDA MB-231 cells cultured in the 6 wells plate were treated with IC_50_ of NLC-Citral and incubated at 37 °C for 48 hours. Total RNA was extracted from the treated cells using the Qiagen RNeasy Kit (Qiagen, USA) according to the manufacturer’s instructions. In brief, cells were lysed in a 500 µL of RLT buffer and then passed through the RNeasy spin column, where total RNA bound to the membrane of the column. Next, the contaminants were removed with 700 µL and 500 µL of RW1 and RPE buffers by flow through the column respectively. Finally, high quality of total RNA was eluted in 50 µL of RNase free water.

### Quality control of RNA

RNA integrity was determined by measuring 28S/18S ratio to calculate the RNA integrity number (RIN). RNA was tested using RNA pico chip (Agilent Technologies, USA). Agilent 2100 bio-analyzer was used to perform the analysis. In addition, the concentration of the extracted total RNA was measured with Qubit Fluorometer (Thermo Fisher Scientific, USA). In addition, the purity of the extracted RNA was measured using a spectrophotometer (Beckman Coulter, USA). High-quality total RNA samples have an A_260/280_ ration of 1.8 to 2.0 that indicates no contaminations of proteins in the sample.

### Microarray-based gene expression analysis

This experiment was used to study the gene expression of MDA MB-231 cells treated with NLC-Citral at IC_50_ for 48 hours whereas NLC-Blank treated MDA MB-231 cells was served as negative control. The experiment was done in triplicate for each group, in which SurePrint G3 Human Gene Expression 8 × 60 K Microarray Kit was used (Agilent Technologies, USA). Briefly, synthesis of cDNA from the total RNA (25 ng) was done using RNA Spike-In Kit (Agilent Technologies, USA). cRNA was synthesis and labeled with Low Input Quick Amp Labeling Kit, one-color cyanine 3 (Agilent Technologies, USA). After that, the RNAeasy Mini Kit (Qiagen, USA)was used to purify the labeled and amplified RNA. The cRNA concentration was then examined using ND-1000 UV-VIS Spectrophotometer (Agilent Technologies, USA). Later on, the cRNA was hybridized onto Agilent SurePrint G3 Human GE 8 × 60 K microarray slide using Microarray Hybridization Chamber Kit (G2534A) (Agilent Technologies, USA) for 17 hours at 65 °C and 10 rpm in oven. Next, the slide was washed with the Gene Expression Wash Buffer 1 and 2 before scanning on Agilent SureScan D (G4900DA) model (Agilent Technologies, USA). Differential expressions in between the treated and untreated cells were then analyzed using GeneSpring GX (Agilent Technologies, USA). In this study, Single Experiment Analysis (SEA) pathway from GeneSpring 13.0 software was applied to explicate putative pathways associated with the differential gene expression in MDA MB-231 cells affected by NLC-Citral and citral.

### Data validation by targeted RNA sequencing (TruSeq)

The targeted RNA sequencing for gene expression study was conducted to measure the expression level of selected genes of interest in MDA MB-231 cells after being treated with NLC-Citral at selected dose. The targeted RNA expression project was first designed using Design Studio software (Illumina, USA). In brief, 75 ng of total RNA was reversed transcribed into the cDNA. The ProtoScript II Reverse Transcriptase and RCS1 were mixed with the total RNA and placed on the pre-programmed thermal cycler for 30 minutes. After that, the hybridization of the oligo pool was done by adding the Targeted Oligo Pool (TOP) mixture to the tube containing cDNA. Next, OB1 was poured and placed on the pre-programmed thermal cycler to anneal. AM1 wash buffer was added to remove the unbound oligos and the supernatant was discarded using the magnetic stand. Then, UB1 was immediately added and the supernatant was removed. Subsequent, the ELM4 was poured and incubated at 37 °C for 45 minutes. For PCR amplification, diluted HP3 was added and incubated for 8 minutes at room temperature. Then, the mixture of TDP1 and PMM2 were spilled and placed on the magnetic stand until the liquid appears clear. Then placed on the thermal cycle for the PCR amplification process. Afterward, the AMPure XP Beads was added to the tube and dispersed completely before the supernatant was transferred into the new tube by using the magnetic stand. About 80% of ethanol was poured into the tube while standing on the magnetic stand without disturbing the beads. The supernatant was discarded, the tube air dried for about 15 minutes and the RSB was inserted before it was placed on the magnetic stand. Finally, the library pooling and quantitation were done in preparation for the sequencing process. Pooled libraries were run on a Miseq instrument (Illumina). All data were analyzed using the Miseq Reporter Targeter RNA application (BaseSpace).

### Data validation by quantitative real-time polymerase chain reaction (qPCR)

Total RNA extracted from the treated cells was converted to cDNA using Maxima First Strand cDNA synthesis kit (Thermo Scientific, USA) and was run into a thermal cycler (Labnet, USA). Then, the qPCR reaction was performed using SYBR Select Master Mix (Life Technologies, USA) in the Eco Illumina (Illumina, USA) to quantify the differential expression of the selected genes. The PCR parameters were, 95 °C for 10 minutes, 40 cycles of 95 °C for DNA denaturation, and 55 °C for 30 seconds. The forward and reverse sequences of the target and housekeeping genes (GAPDH and ACTB) were obtained from Primer-Blast NCBI (Table 3). The quantity of the genes was calculated and measured by Eco study software (Illumina, USA). The expression levels of NLC-Citral and citral were compared to the NLC-Blank control group.

**Table 3:** List of gene name, accession number, and sequence of the primers used in the real-time qPCR analysis.

Gene Name	Accession Number	Sequence of Primers
SNAIL	NM_013599.3	F: 5′-GCCGACTTTTGTGGTCTTCC-3′ R: 5′-GGTACAAGTATGCCTCTGCCA-3′
PLK-1	NM_010927.3	F: 5′-GCACCGAGATTGGAGTTC-3′ R: 5′-GAGCACAGCCACATTGAT-3′
NF-κB	NM_0086892	F: 5′-CCTGCTTCTGGAGGGTGATG-3′ R: 5′- GCCGCTATATGCAGAGGTGT -3′
CDKN1B	NM_010849.4	F: 5′-TGATGTGGTGTCTTGGAGAA-3′ R: 5′-CGTAGTTGTGCTGGTGAGTG-3′
GAPDH	NM_008084.3	F: 5′-GAAGGTGGTGAAGCAGGCATC-3′ R: 5′-GAAGGTGGAAGAGTGGGAGTT-3′
CTB	NM_007393.3	F: 5′-TTCCAGCCTTCCTTCTTG-3′ R: 5′-GGAGCCAGAGCAGTAATC-3′

### Proteome profiling of apoptosis-related proteins

The expression profile of apoptosis-related proteins was investigated on the MDA MB-231 cells treated with NLC-Citral for 48 hours using the Human Apoptosis Array Kit (R & D Systems, USA). Protein from the cells was extracted using 600 µL of RIPA buffer (50 mM Tris-HCl, 150 mM NaCl, 1.0% TritonX-100, 0.5% sodium deoxycholate, and 0.1% SDS) supplemented with 10 mg of protease inhibitor cocktails (Roche, Canada). The protein was then quantified using Bradford reagent (Sigma, USA). Concisely, the membrane was incubated for 1 hour in the 2.0 mL array buffer 1 and the protein sample was prepared with the addition of 1.0 mL lysis buffer 17 to the protein lysate. Next, prepared sample was loaded into the membrane and incubated at 4 °C overnight. Then, the membrane was washed 3 times with 20 mL wash buffer, transferred into the 4-well multi-dish containing detection antibody cocktails and incubated for 1 hour. Two mL diluted Streptavidin-HRP with array buffer was poured, incubated for 30 minutes,and washed 3 times before 1.0 mL of Chemi Reagent Mix was pipetted onto the membrane for viewing. The membrane was scanned using the ChemiDoc XRS (Bio-Rad, USA).

### Data validation by Enzyme-Linked Immunosorbent Assay (ELISA)

The quantified serum proteins including survivin, Cytochrome C, Bax and Bcl-2 concentrations were determined in triplicates and validated using the quantitative human colorimetric ELISA kit (Abcam, USA). Then, relative expression was calculated by dividing sample against NLC-Blank and a standard curve was generated for each protein separately.

### Animals

Female adult BALB/c mice aged 6 to 8 weeks were purchased from the Animal House of Faculty of Veterinary, University Putra Malaysia, Serdang (UPM, Malaysia). The mice were acclimatized for one week and housed under the standard condition at 24° ± 1 °C under 12-hours dark and light cycle. The mice were provided pellet and water ad libitum during the whole period of study. This study was approved by the Animal Care and Use Committee, Faculty of Veterinary Medicine, University Putra Malaysia (UPM/IACUC/AUP-R098/2014).

### Animals grouping and treatment

The mice were grouped into 3 groups (n = 6 for each group): (1) NLC-Blank group, (2) NLC-Citral treated group, and (3) Citral treated group. Mice in all 3 groups were inoculated with 1 × 10^5^ 4T1 cells in RPMI-1640 media via subcutaneous injection. All treated mice were fed orally with NLC-Citral and citral with 50 mg/kg daily using oral gavage for 28 days starting on the first day of inoculation. The dose was selected based on the studies conducted previously^85^. Whereas, the negative control group was treated with the vehicle used to encapsulate the citral (NLC-Blank).

### Measuring the tumor weight and size

The 4T1 tumor harvested from the mice after 28 days of treatment with NLC-Blank, NLC-Citral, and citral were weighted using a weighing balance (Mettler Toledo, Switzerland) while the tumor size were measured using a caliper and the tumour volumes were calculated using the formula V = 1/2 (width2 × length).

### Statistical analysis

All experiments were done in triplicates and the average values were obtained. The statistical analysis was performed using the GraphPad Prism 6.0. One way ANOVA was selected for the experimental analysis with Tukey’s post hoc test. The significance was set at p < 0.05 when comparing to NLC-Blank as a control and NLC-Citral and citral as treated groups.
